# Comprehensive Review of the Imaging Recommendations for Diagnosis, Staging, and Management of Thyroid Carcinoma

**DOI:** 10.3390/jcm13102904

**Published:** 2024-05-14

**Authors:** Nivedita Chakrabarty, Abhishek Mahajan, Sandip Basu, Anil K. D’Cruz

**Affiliations:** 1 Department of Radiodiagnosis, Advanced Centre for Treatment, Research and Education in Cancer (ACTREC), Tata Memorial Centre, Homi Bhabha National Institute (HBNI), Parel, Mumbai 400012, Maharashtra, India; dr.niveditachakrabarty@gmail.com; 2 Department of Imaging, The Clatterbridge Cancer Centre NHS Foundation Trust, 65 Pembroke Place, Liverpool L7 8YA, UK; 3 Faculty of Health and Life Sciences, University of Liverpool, Liverpool L69 3BX, UK; 4 Radiation Medicine Centre, Bhabha Atomic Research Centre, Tata Memorial Hospital Annexe, Homi Bhabha National Institute (HBNI), Parel, Mumbai 400012, Maharashtra, India; drsandipb@gmail.com; 5 Apollo Hospitals, Navi Mumbai 400614, Maharashtra, India; docdcruz@gmail.com; 6 Foundation of Head Neck Oncology, Mumbai 400012, Maharashtra, India; 7 Union International Cancer Control (UICC), 1202 Geneva, Switzerland

**Keywords:** thyroid carcinoma, ultrasound, cross-sectional imaging, risk factor and etiopathogenesis, American Thyroid Association management guidelines, radioiodine scan

## Abstract

Thyroid cancer is the most common head and neck cancer (HNC) in the world. In this article, we comprehensively cover baseline, posttreatment, and follow-up imaging recommendations for thyroid carcinomas along with the eighth edition of the tumor, node, metastasis (TNM) staging system proposed by the American Joint Committee on Cancer (AJCC) and the Union for International Cancer Control (UICC). We include characterization and risk stratification of thyroid nodules on ultrasound (US) proposed by various international bodies. Management guidelines (depending upon the type of thyroid carcinoma) based on the international consensus recommendations (mainly by the American Thyroid Association) are also extensively covered in this article, including the role of a radioiodine scan. The management of recurrent disease is also briefly elucidated in this article. In addition, we cover the risk factors and etiopathogenesis of thyroid carcinoma along with the non-imaging diagnostic workup essential for thyroid carcinoma management, including the significance of genetic mutations. US is the diagnostic imaging modality of choice, with US-guided fine needle aspiration (FNA) being the procedure of choice for tissue diagnosis. The roles of computed tomography (CT), magnetic resonance imaging (MRI), and fluorodeoxyglucose positron emission tomography/CT (FDG-PET/CT) in thyroid carcinoma staging are also specified. Through this article, we aim to provide a comprehensive reference guide for the radiologists and the clinicians in the pursuit of optimal care for patients with thyroid carcinoma.

## 1. Introduction

Thyroid cancer is the most common head and neck cancer and is responsible for 3% of new cancers worldwide (ranked 10th) [1]. Papillary thyroid carcinoma (PTC) is the most common (88%), followed by follicular thyroid carcinoma (FTC) (8%), medullary thyroid carcinoma (MTC) (2–5%), poorly differentiated thyroid carcinoma (PDTC) (6%), and anaplastic thyroid carcinoma (ATC) (1%) [2,3,4,5]. Based on the 2022 World Health Organization (WHO) classification of thyroid neoplasms, malignant thyroid neoplasms are categorized into: FTC, invasive encapsulated follicular variant of PTC, PTC, oncocytic carcinoma of the thyroid, differentiated high-grade thyroid carcinoma, PDTC, and ATC; low-risk thyroid neoplasms are categorized into: non-invasive follicular thyroid neoplasm with papillary-like nuclear features, follicular thyroid tumor of uncertain malignant potential, well-differentiated thyroid tumor of uncertain malignant potential, and hyalinizing trabecular thyroid tumor [6]. The 2022 WHO classification endorses histological grading of MTC into high-grade and low-grade lesions [6]. Papillary cancer ≤ 1 cm is referred to as papillary microcarcinoma (PMC) irrespective of whether high-risk features are present or not [7,8]. PTC, FTC, poorly differentiated and anaplastic carcinomas arise from follicular cells in the thyroid gland, of which PTC and FTC are the differentiated thyroid carcinomas (DTC) [3,9]. The origin of medullary thyroid carcinoma (MTC) is the parafollicular C cells of the thyroid gland [10].

Imaging plays a key role in detecting, localizing, and characterizing thyroid malignancy, as well as in pre-surgical planning, detecting lymph nodes and distant metastasis, and identifying recurrence. In this article, we comprehensively cover the risk factors, etiopathogenesis, and the standard diagnostic (imaging and non-imaging) and management recommendations pertaining to thyroid carcinomas, including management of recurrence. The roles of cross-sectional imaging (computed tomography (CT) and magnetic resonance imaging (MRI) and fluorodeoxyglucose positron emission tomography (FDG PET) CT, in addition to ultrasound, for thyroid cancer management are extensively covered in this article. The purpose of our article is to keep the readers (radiologists and clinicians) updated about the existing international guidelines for the management of thyroid carcinoma and also to highlight the potential areas of future research.

## 2. Risk Factors and Clinical Presentations

More than 90% of thyroid carcinomas are sporadic in nature [11]. A history of radiation therapy (RT) for HNC in childhood, total body radiation for bone marrow transplantation, and ionizing radiation exposure from fallout during childhood or adolescence are the risk factors for PTC [3,12,13]. A family history of thyroid carcinoma, independently or associated with thyroid carcinoma syndromes, are risk factors for DTC and MTC [3]. Type 2 multiple endocrine neoplasia (MEN) syndromes (MEN2A and MEN2B) and familial MTC (FMTC) are risk factors for MTC [3]. Cowden syndrome, familial adenomatous polyposis, Carney complex, and Werner syndrome are risk factors for DTC [14]. Low iodine content in the diet is linked to follicular and anaplastic thyroid carcinomas [3]. Anaplastic thyroid carcinoma (ATC) can also arise from pre-existing PTC in those with TERT promoter mutation [15]. Patients usually present with neck swelling and hoarseness of voice.

## 3. Epidemiology and Etiopathogenesis

Thyroid carcinoma is more commonly diagnosed in women than men [14,16]. DTC predominantly occurs between the ages of 25 and 54 years, though any age group can be affected [17]. DTC in elderly patients has a worse prognosis and a high recurrence rate due to aggressive histopathology and delay in diagnosis; hence, enhanced vigilance by the physician is warranted [18]. Sporadic MTC is seen between 40 and 60 years of age [19]. ATC is usually diagnosed at ≥65 years of age [20].

The activation of mitogen-activated protein kinase (MAPK) is essential for PTC initiation through point mutations of BRAF and RET (rearranged during transfection) genes [21]. Phosphatidylinositol-3 kinase (PI3K)/AKT activation triggered by activating mutations in RAS (rat sarcoma virus), PIK3CA, and AKT1 and the inactivation of the phosphatase and tensin homolog (PTEN) play a crucial role in the initiation of FTC [21]. MTC originates from neuroendocrine C cells that produce calcitonin within the thyroid gland [3]. Almost all patients (98%) with MEN 2A, MEN 2B, and FMTC show RET germline mutations, whereas somatic RET mutations are seen in approximately 45–70% of sporadic MTC [19,22,23,24,25]. TERT promoter mutation is common in PDTC and ATC and is associated with increased risk of distant metastases and death. ATC also frequently shows TP53 mutation, which differentiates it from PDTC [21].

PTC commonly shows regional nodal metastases and can present with multicentric thyroid nodules, whereas FTC has an increased propensity for distant organ metastasis compared to regional nodal metastasis [26]. Classic PTC has a good prognosis, whereas variants of PTC, such as tall cell, hobnail, solid, and columnar, show aggressive clinical behavior [27].

Approximately 48% of MTCs have localized disease at initial presentation; 35% have extrathyroidal extension (ETE) and regional nodal metastasis; and 13% have distant metastasis to lungs, liver, or bones [10,28,29]. ATC is highly invasive with a high incidence of extrathyroidal, lymphatic, and vascular extensions and an increased propensity for distant metastasis [15].

## 4. Imaging Referral Guidelines

The American Thyroid Association (ATA), American Association of Clinical Endocrinologists (AACE), National Comprehensive Cancer Network (NCCN), and European Society of Medical Oncology (ESMO) have laid down management recommendations for thyroid carcinoma, of which the ATA guidelines are commonly practiced [14,15,19,30,31,32]. The characterization and risk stratification of thyroid nodules on US, with further guidelines on fine needle aspiration (FNA) and the follow-up, have been proposed by various international bodies, including the ATA; American College of Radiology Thyroid Imaging Reporting and Data Systems (ACR TI-RADS); TI-RADS released by the Korean Society of Thyroid Radiology (K-TIRADS); TIRADS developed by Horvath et al. [33]; European Thyroid Association (EU-TIRADS); British Thyroid Association (BTA); TIRADS developed by Kwak et al. [34]. (Kwak-TIRADS); Society of Radiologists in Ultrasound (SRU); AACE; American College of Endocrinology (ACE) and Associazione Medici Endocrinologi (AME) Medical Guidelines for Clinical Practice for the Diagnosis and Management of Thyroid Nodules; French (F)-TIRADS; and Thyroid Multimodal-imaging Comprehensive Risk Stratification Scoring (TMC-RSS) (Table 1) [30,33,34,35,36,37,38,39,40,41].

## 5. Clinical/Non-Imaging Diagnostic Workup

Physical examination is warranted to detect palpable thyroid nodule and cervical lymphadenopathy. Fixation of the thyroid nodule to surrounding tissues in the neck on physical examination is indicative of thyroid carcinoma.

Serum thyrotropin (TSH) measurement should be performed for all patients with a >1 cm sized thyroid nodule [14]. Diagnostic FNA is performed based on the recommendations of the ultrasound (US) risk stratification system used, and the Bethesda System for Reporting Thyroid Cytopathology should be followed for evaluating thyroid nodule FNA [14,42]. Thyroid core needle biopsy and seven gene mutation marker panels (BRAF, NRAS, HRAS, KRAS, RET/PTC1, RET/PTC3, and PAX8/PPARγ) may be considered in those with indeterminate FNA cytology and in those with suspicious PTC cytology if it is expected to alter surgical decisions [14].

Serum calcitonin and carcinoembryonic antigen (CEA) should be measured in those suspected of having MTC, and a markedly elevated CEA out of proportion to calcitonin indicates aggressive MTC [19]. Elevated serum calcitonin, chromogranin, and CEA on immunohistochemistry (IHC) and the absence of thyroglobulin staining suggest a diagnosis of MTC [19]. Those suspected of having MEN2A should undergo direct DNA analysis to detect *RET* mutations in exon 10 (codons 609, 611, 618, and 620), exon 11 (codons 630 and 634), and exons 8, 13, 14, 15, and 16, whereas patients with the MEN2B phenotype should be tested for *RET* codon M918T mutation (exon 16) and, if negative, the *RET* codon A883F mutation (exon 15). Genetic testing to detect germline RET mutation should also be offered to patients with apparent sporadic MTC, as hereditary disease may be seen in 1–7% of presumed sporadic MTC [19,43,44,45].

The diagnosis of ATC is established by FNA biopsy and the analysis of IHC markers on the cell block if the aspirate is cellular; otherwise, core biopsy may be required [12,13,14,15]. ATC can co-exist or occur in patients with resected DTC. IHC markers that suggest a diagnosis of ATC include *BRAF^V600E^* (specific and sensitive), Ki-67 > 30%, PAX8 (retained in 40–60%), and the somatic mutation of TP53 [15]. Additionally, complete blood count, serum electrolytes, serum calcium, blood urea nitrogen, creatinine, blood glucose, a liver function test, and a thyroid function test should form a part of the preliminary investigations for ATC. The expression of thyroglobulin is retained in the majority of PDTCs, differentiating it from ATC [15].

## 6. Imaging Guidelines

### 6.1. Diagnosis

US is the investigation modality of choice for confirming the presence of a thyroid nodule incidentally detected on other modalities (CT/MRI/FDG PET CT), for characterizing a thyroid nodule, and to rule out metastatic cervical lymph nodes, particularly lateral compartment nodes [14,19,46]. It has been observed that CECT has a better accuracy than US for the assessment of central compartment nodes due to technical challenges posed by the overlying thyroid, clavicle, and sternocleidomastoid muscle when using US for central compartment nodal evaluation [47,48]. Diagnosis is established using US-guided FNA. Aggressive variants of PTC, such as tall cell, hobnail, solid, and columnar, are frequently associated with ETE, lymph nodal, and distant metastasis [49,50,51].

#### 6.1.1. US of Thyroid

**Figure 1:**
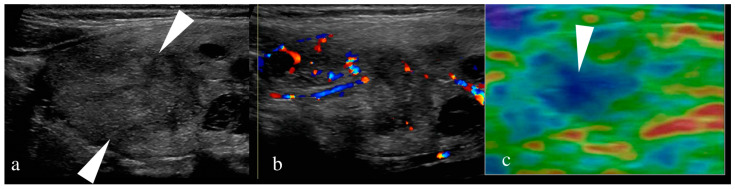
(**a**–**c**): (**a**) Ultrasound of thyroid shows a 3.2 cm solid, hypoechoic nodule (arrowhead), wider than taller with micro-lobulated margin without any micro-calcifications. (**b**) The nodule shows internal vascularity on color doppler image. (**c**) Elastography of the nodule shows hard consistency seen as blue colour (arrowhead). In colour elastogram, tissue hardness increases in ascending order from red, yellow, green and blue.

A high-frequency linear array probe (7–15 MHz) is used to scan the patient in a supine position with their neck hyperextended [43,44,46,47,49,50,51,52]. All the US thyroid nodule risk stratification systems recommend evaluation of the thyroid nodule based on echogenicity, shape, margin, and presence or absence of echogenic foci on gray scale US [30,35,36,37,38,39,40,53]. BTA, the AACE/ACE/AME guidelines, SRU, TIRADS developed by Horvath et al., and TMC-RSS additionally use the vascularization pattern to characterize the nodule, and F-TIRADS, the AACE/CE/AME guidelines, and TMC-RSS also have provisions to include sonoelastography for thyroid nodule characterization (Figure 1) [30,40,54].

As per the 2015 ATA guidelines, US findings with a high suspicion of malignancy (>70–90%) include a completely solid hypoechoic nodule, a solid hypoechoic component of a partially cystic nodule that has one or more of the features, such as irregular margins (infiltrative, microlobulated), microcalcifications, a taller-than-wide shape, rim calcifications with a small extrusive soft tissue component, and the presence of ETE [14]. Some of the studies comparing the various US-based thyroid nodule risk stratifications are shown in Table 2 [39,55,56,57,58,59]. In addition, the thyroid gland should also be evaluated for underlying diffuse inflammatory conditions, like Hashimoto’s thyroiditis.

#### 6.1.2. US of Neck Nodes

US evaluation of the neck from submental region to sternal notch for cervical nodal metastasis is an essential component of thyroid US examination. In addition to thyroid nodule characteristics, cervical lymph node status is also incorporated in the AACE/CE/AME guidelines, F-TIRADS, BTA, ATA, K-TIRADS, and TMC-RSS [30,40]. Nodal metastasis from thyroid cancer is common in the central compartment (level VI) and lateral group of nodes (levels II to IV) [60]. PTC thyroid shows a high incidence of nodal metastasis, ranging from 30 to 90%. The incidence rates of nodal metastasis from MTC, ATC, and FTC are approximately 50%, 40%, and 10%, respectively [60,61]. US features predictions of nodal metastasis, including microcalcifications, cystic components, peripheral vascularity, hyperechogenicity, round shape, loss of fatty hilum, and extranodal extension (ENE) [14,60]. Hyperechogenocity, microcalcifications, and cystic components are common in nodal metastasis from PTC, whereas necrosis and ENE are common from ATC [60,62,63].

#### 6.1.3. US-Guided FNA of Thyroid Nodule/Neck Nodes

FNA of the thyroid nodule should be conducted based on the US risk stratification criteria and FNA should also be performed from any suspicious cervical lymph nodes [30,35,36,37,38,39,40]. As shown in Table 2, unnecessary FNAs are reduced significantly by using ACR-TIRADS [39,58]. When ≥3 thyroid nodules qualify for biopsy as per the ACR TI-RADS guidelines, the two most suspicious should be biopsied [35]. If serum TSH in a thyroid nodule >1 cm in size is subnormal, then, additionally, the patient should be subjected to a radionuclide (preferably ^123^I) thyroid scan, and its findings compared with US features; only hypofunctioning nodules which meet the US criteria for FNA should be biopsied [14]. Prior to performing FNA biopsy, informed consent of the patient should be obtained. After localizing the nodule on US, the overlying skin is cleansed with a 10% povidone-iodine solution. The skin and superficial subcutaneous tissue overlying the nodule may be injected with approximately 1–2 mL of 1% lidocaine hydrochloride solution [64]. Thyroid FNA is performed using a 23–27-gauge needle [64]. A parallel technique is used for superficial location of the nodule, and the needle is visualized in its entirety with this technique. For a nodule situated deep within the thyroid gland, a perpendicular technique is used and only the tip of the needle is visualized with such a technique [64]. The tip of the needle should be placed at the center of the nodule/neck node being biopsied. The 3–4 needle passes with either the capillary (to and fro movement of the needle within the nodule/node without suction) or the aspiration technique (with suction), is sufficient, if an on-site cytopathologist for evaluation of the adequacy is not available [65]. For a core needle biopsy in the case of a non-diagnostic FNA, an 18–20-gauge needle should be used [64]. Thyroglobulin (Tg) estimation in the washout fluid from lymph node FNA biopsy can provide preoperative information about nodal metastasis and also has an added value in FNA biopsy [66,67].

### 6.2. Staging

Presently, the eighth edition of the tumor, node, metastasis (TNM) staging system for thyroid cancer proposed by the American Joint Committee on Cancer (AJCC) and the Union for International Cancer Control (UICC) is being utilized, as shown in Table S1 [68,69]. Though the TNM descriptors for differentiated carcinoma and ATC are the same, the prognostic stage groups are different as all the ATCs are categorized as stage IV (A-C) [68]. Table S2 shows the stage groups for DTC, ATC, and MTC [68,69]. For extensive and invasive disease or clinically obvious neck nodes, contrast-enhanced computed tomography (CECT) or CE magnetic resonance imaging (MRI) of the neck is recommended as an additional investigation as per the 2015 ATA guidelines for DTC [14]. The imaging recommendations for staging MTC and ATC based on the ATA guidelines are mentioned in Table 3 [15,19].

ETE of thyroid carcinoma to crucial structures such as trachea, esophagus, carotid, or mediastinal vessels, may entail major reconstructive surgeries or render the patient inoperable, and since US has limitations in evaluating these structures, cross-sectional imaging (CT/MRI) is essential for pre-surgical planning [60].

**Figure 2:**
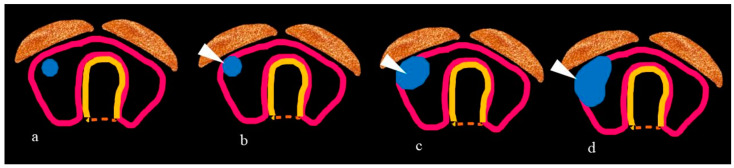
(**a**–**d**): Pictorial illustration of grades of extrathyroid extension (ETE). (**a**) Grade 0 ETE—a tumor (in blue) entirely surrounded by thyroid parenchyma. (**b**) Grade I ETE—a tumor in which 1–25% (arrowhead) of the tumor margin is in contact with the thyroid capsule (**c**) Grade II—a tumor in which 25–50% (arrowhead) of the tumor margin is in contact with the capsule. (**d**) Grade III—a tumor (arrowhead) in which >50% of the tumor margin is in contact with the capsule. Colour coding: Blue = Tumour, Dark pink outlined structure = Thyroid gland, Yellow = Tracheal cartilage, Orange = Strap muscle. (Adapted from [71]).

ETE can be categorized from grade 0 to grade III based on the contact of the tumor with and the disruption of the thyroid capsule, as pictorially depicted in Figure 2, and CECT outperforms US in ETE grading for tumors having >50% capsular contact [70].

**Figure 3:**
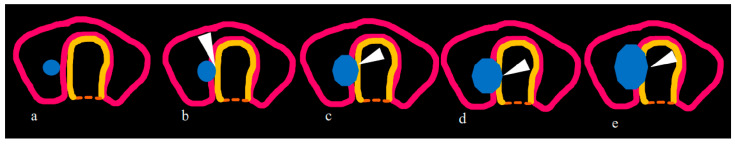
(**a**–**e**): Pictorial illustration of modified SHIN grading for tracheal invasion: (**a**) Grade 0: >5 mm distance between tumor (in blue) and tracheal cartilage (in yellow). (**b**) Grade I: disease abuts external perichondrium (arrowhead). (**c**) Grade II: disease invades into the cartilage (arrowhead) with/without destruction. (**d**) Grade III: disease extends into the tracheal mucosa (arrowhead) without any elevation or infiltration of mucosa. (**e**) Grade IV: disease shows full-thickness invasion with elevation and bulging of the tracheal mucosa (arrowhead). Colour coding: Blue = Tumour, Dark pink outlined structure = Thyroid gland, Yellow = Tracheal cartilage. (Adapted from [71]).

A tumor posterior to the trachea in the midline is a “blind spot” for US and requires evaluation using cross-sectional imaging [60,61]. Effacement of fat in the tracheoesophageal groove or between the laryngeal cartilage and hypopharyngeal wall suggests tumor extension on CT and MRI [71]. Tracheal invasion on CT is evaluated using the SHIN grading, as pictorially depicted in Figure 3, which helps in pre-surgical planning, as a shave procedure is sufficient in early involvement and requires segmental resection in the case of extensive involvement [71,72].

CECT is pertinent for documenting the aberrant origin of the right subclavian artery, in which case there is a non-recurrent inferior laryngeal nerve; a variant of the inferior laryngeal nerve, that has to be borne in mind before operating on such cases [71]. Owing to its high soft tissue contrast, MRI better delineates the tumor invasion of the strap muscle, larynx, and esophagus and the infiltration of the marrow [3,71,73]. The predictive value of MRI for esophageal layer involvement is 82% for the outer layer and 100% for the inner layer [74]. CT has high specificity (96.2%) but lacks sensitivity for esophageal involvement [74,75].

Table 4 shows adjacent structure encasement criteria for thyroid carcinoma on CT/MRI [74,75,76,77,78,79,80].

There are no established guidelines regarding the minimum gap between CECT with iodinated contrast agents and iodine-131/123 for whole body scintigraphy (WBS) in the treatment of residual disease and distant metastases, but the majority recommend a gap between 4 weeks and 2 months [32,81,82,83].

Imaging findings and their implications in the management of thyroid carcinoma are shown in Table 5 [71].

Table 6 shows a few of the cross-sectional imaging-based studies for baseline evaluation of thyroid cancer and neck nodes in the last 15 years [48,76,84,85,86,87].

Synoptic CT reporting template for thyroid carcinoma is attached in Figure S1 [71].

**Figure 4:**
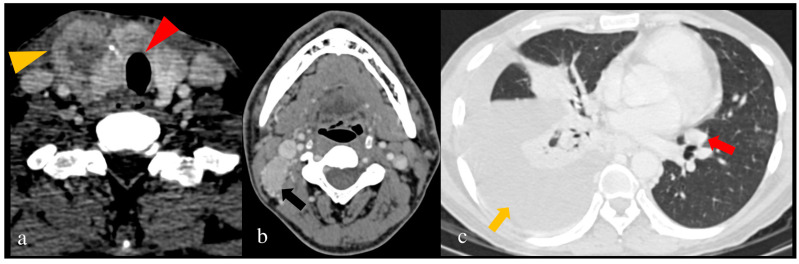
Stage II papillary carcinoma thyroid in a 40-year-old male patient shows: (**a**) extrathyroidal extension (yellow arrowhead) and tracheal cartilage involvement without tracheal mucosal involvement (SHIN II (red arrowhead)) (**b**) metastatic right level II node (black arrow), (**c**) metastatic right pleural effusion (yellow arrow), and metastatic left lung lower lobe nodule (red arrow) on contrast-enhanced computed tomography (CECT).

Figure 4 shows CT scan of a thyroid carcinoma patient with ETE and lung metastasis.

## 7. Principles of Management

Surgery is the mainstay of treatment for thyroid carcinoma [14,15,19]. The 2009 ATA guidelines, which were subsequently modified in 2015, advocate a risk stratification approach for DTC (low-, intermediate-, and high-grade), based on age, gender, tumor size with extension, lymph node involvement, and distant metastasis, to identify those at a high risk of mortality who require more aggressive surgical and adjuvant treatment [88,89].

### 7.1. Radioactive Iodine (RAI) Scan

**Figure 5:**
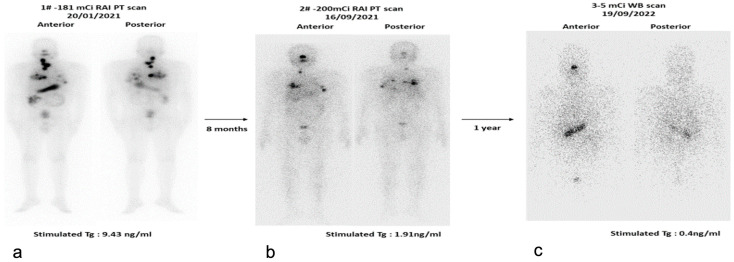
(**a**–**c**). A 32-year-old male with differentiated papillary thyroid carcinoma, classical type with lymph node and bilateral lung metastases. He underwent total thyroidectomy bilateral nodal dissection on 26 December 2019 and was considered for adjuvant radioiodine therapy post-surgery. The 1st post-treatment scan (**a**) shows abnormal radioiodine concentration in thyroid bed, adjacent nodes, and bilateral lungs. The 2nd post-treatment scan (**b**) demonstrated gradual resolution of lymph nodal uptake and partial response of bilateral lung lesions. The diagnostic scan after 1 year of 2nd therapy (**c**) shows complete resolution of all lesions. The tumor marker-stimulated Tg showed serial decrease from 9.43 ng/mL (pre-therapy) to 0.4 ng/mL (1 year after 2nd therapy).

Both I-131 and I-123 are routinely used for imaging and assessment of functional thyroid tissue and thyroid cancer remnant/metastasis following total thyroidectomy [14,90,91]. Figure 5 depicts the importance of I-131 theranostics in DTC with locoregional and distant metastases (high-risk group).

The addition of a diagnostic ^131^I whole body scan (WBS) in the treatment strategy for intermediate and high-risk thyroid cancer enables a disease survey and guides the ^131^I therapeutic administration. The enhancement of image acquisition parameters and current SPECT/CT gamma camera technology allow high-quality visualization of locoregional disease and distant metastatic disease using 37 MBq (1 mCi) ^131^I diagnostic activity [91]. In all patients who receive ^131^I therapy, post-therapy (PT) WBS is usually performed after 2–10 days, during discharge from the isolation ward when the exposure rate reduces below the limits.

### 7.2. Role of FDG-PET/CT

As per the recently published Society of Nuclear Medicine and Molecular Imaging (SNMMI).

**Figure 6:**
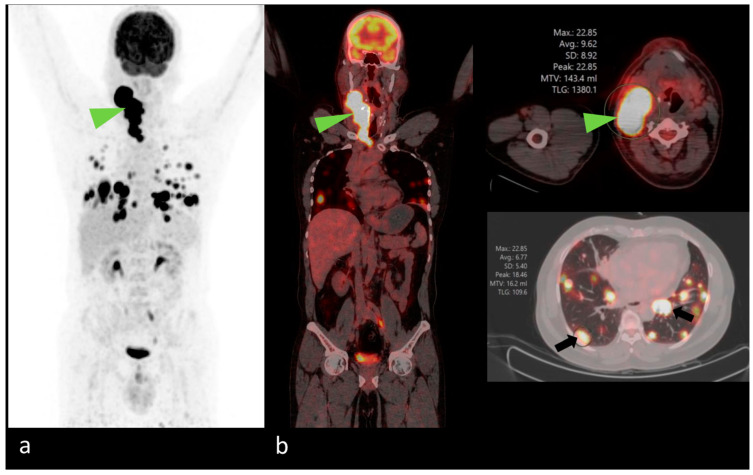
(**a**,**b**): Known case of sarcomatoid carcinoma of thyroid with anaplastic component, following total thyroidectomy and neck dissection in May 2023. The patient now complains of increase in right-sided neck swelling from 2 weeks. ^18^F-fluorodeoxyglucose positron emission tomography/CT (FDG-PET/CT) scan performed for disease status evaluation reveals metabolically active conglomerated mass of enlarged right cervical lymph nodes, level (II–IV) (arrowheads in maximum intensity projection (MIP) images (**a**) and fused PET-CT images (**b**)) with metastatic multiple enlarged nodules scattered in bilateral lung parenchyma (arrows in (**b**)).

The Procedure Standard/European Association of Nuclear Medicine (EANM) Practice Guidelines, ^18^F-FDG PET/CT, offer potential clinical benefits in the management of aggressive DTC, PDTC, and ATC and can be performed preoperatively in more aggressive DTC histology (i.e., PDTC or Hurthle cell carcinoma) and ATC (Figure 6) [91].

**Figure 7:**
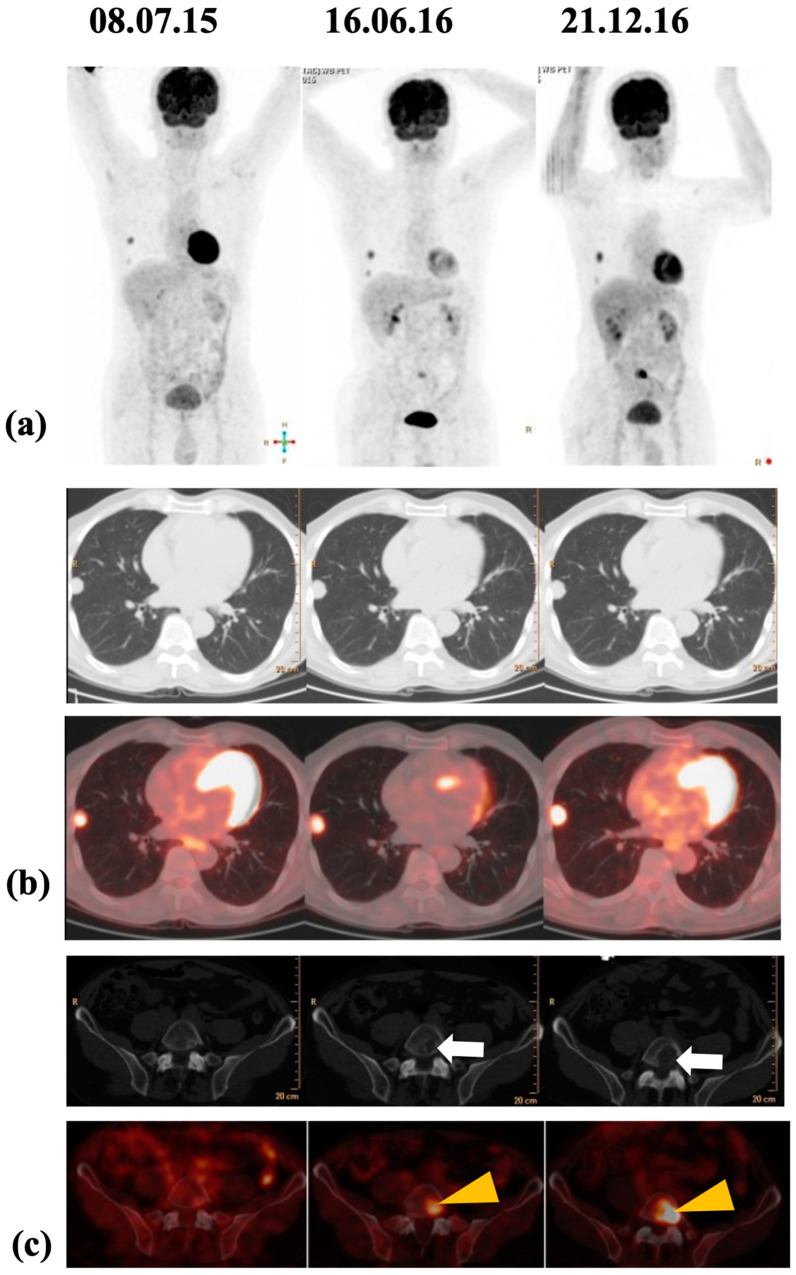
(**a**–**c**). Comparative ^18^F-fluorodeoxyglucose positron emission tomography/CT (FDG-PET/CT) maximum intensity projection (MIP) images (**a**), transaxial CT and fused PET-CT images of the lung lesions (**b**), and transaxial CT and fused PET-CT images of the L5 vertebral lesion (**c**) undertaken at 3 different time points of the disease course. While the bone metastasis is not a criterion in the standard RECIST criteria, the progressive disease was clearly evident in increasing indices on FDG-PET (arrowheadss) and osteolysis on CT (arrows). Reproduced with permission from: [92] 2017, Springer-Verlag Berlin Heidelberg.

In the post-operative follow-up scenario, ^18^F-FDG PET/CT can be used to identify lesions in patients in whom there is a suspicion of non-iodine–avid metastatic disease (based on elevated basal and/or stimulated Tg and negative radioiodine scan, i.e., Tg+/scan), classically termed as ‘TENIS’. Figure 7 shows a TENIS patient exemplified with temporal profile of FDG-PET/CT [92] Following systemic therapies (such as tyrosine kinase inhibitors in TENIS), FDG-PET/CT is used routinely for treatment response and disease status evaluation. The ^18^F-FDG-PET/CT metabolic parameters (e.g., standardized uptake value (SUV), metabolic tumor volume (MTV), and total lesion glycolysis (TLG)) can be of potential help in defining the biology of the metastatic tumor burden, especially in patients with less favorable or non-response settings (“radioiodine refractory” thyroid cancer) [14,93]. Dedifferentiation of DTC results in an increase in FDG avidity and loss of radioiodine uptake, which is known as the “flip-flop phenomenon” and represents more aggressive disease [94,95].

**Figure 8:**
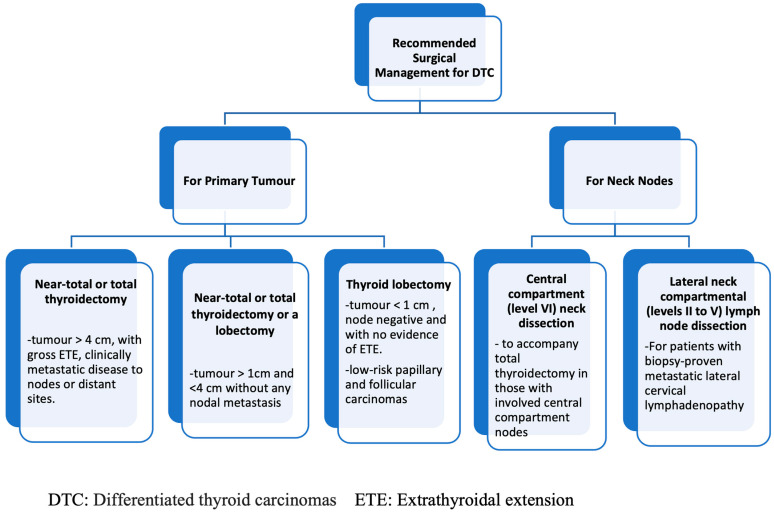
Flowchart depicting surgical management recommendations for differentiated thyroid carcinomas based on the American Thyroid Association guidelines.

2015 ATA management guidelines for DTC: RAI adjuvant therapy is routinely recommended for high-risk DTC patients [14]. A postoperative Tg > 5–10 ng/mL in ATA low- or intermediate-risk DTC patients also warrants RAI ablation [14]. Surgical management recommendations based on ATA are depicted in the flowchart in Figure 8. Recurrent laryngeal nerve (RLN) should be visually identified during dissection, and the external branch of the superior laryngeal nerve (EBSLN) should be preserved during dissection of the superior pole of the thyroid gland. The parathyroid gland along with its vascular supply should be preserved during thyroid dissection. RAI therapy is used for treating pulmonary micrometastases, and RAI therapy improves survival of iodine-avid bone metastases. In the case of symptomatic distant metastasis or a high risk of local complications, consideration should be given to stereotactic radiation or thermal ablation before initiation of systemic therapy [14,96,97].

2015 ATA management guidelines for MTC: Total thyroidectomy with central compartment neck dissection should be performed for patients without neck node metastasis on US and without any distant metastasis. Patients with a positive ipsilateral neck node should also undergo contralateral neck dissection if the serum calcitonin level is >200 pg/mL. Completion thyroidectomy should be performed when RET germline mutation is detected in a patient who has undergone hemithyroidectomy for initially presumed sporadic MTC. Normal parathyroid glands and their vascular supply should be conserved during thyroid dissection. For locally advanced and metastatic MTC, external beam radiotherapy (EBRT), systemic medical therapy, and other nonsurgical therapies (thermoablation, radiofrequency ablation, cryotherapy, chemoembolization) should be considered after a multidisciplinary tumor board discussion. Progressively increasing multiple metastases should be treated with systemic therapy [19,98].

2021 ATA management guidelines for ATC: Surgical resection may be considered for IVA and IVB patients with the aim of achieving R0/R1 resection, followed by intensity modulated radiotherapy (IMRT) and concurrent systemic therapy for those without distant metastasis. For those with R2 resection or unresectable non-metastatic disease, standard fractionation IMRT with systemic therapy may be considered, or combined BRAF/MEK inhibitors can be given to those with *BRAF^V600E^*-mutated ATC, provided the performance status is good. Surgical resection can also be considered if the tumor becomes potentially resectable after RT/and or systemic therapy. For *BRAF* nonmutated patients, radiation therapy with concurrent chemotherapy should be considered. In stage IVC patients with *NTRK* or *RET* fusion, a TRK inhibitor or RET inhibitor in a clinical trial setting (if possible) should be initiated. In IVC patients with high PD-L1 expression, checkpoint (PD-L1, PD1) inhibitors (immunotherapy) can be offered [15]. The best supportive care is to be considered for those with metastatic and progressive ATC [15].

## 8. Follow-Up

As per the ESMO guidelines, DTC patients should undergo a US neck, Tg, and Tg antibody (TgAb) assay 6–18 months after treatment completion (surgery plus radioactive iodine therapy) as part of the follow-up protocol, in which suspicious thyroid bed lesions can be identified on USG, and rising Tg levels are suggestive of residual/recurrent disease [32].

Follow-up guidelines recommended by ATA: posttreatment whole body RAI scan with/without single-photon emission computed tomography (SPECT)/CT after RAI remnant ablation/treatment to record any structural disease with RAI avidity [14,99]; serial Tg measurement to identify patients with residual or recurrent disease and high-risk DTC patients with increased serum Tg (>10 ng/mL); and negative RAI imaging to undergo FDG PET scanning [14]. Initially, serum Tg estimation should be performed every 6–12 months for those on thyroxine therapy, with increased frequency in ATA high-risk patients [14]. After surgery, neck US should be performed at 6–12 months and then at regular intervals for the evaluation of thyroid bed and central and lateral nodal compartments, depending upon the patient’s risk for recurrent disease and Tg status [14].

Mass in the thyroid bed (between carotid and trachea) showing calcification, cystic components, or disorganized vascularity are consistent with recurrence on US. Similarly, rounded nodes with absence or disruption of normal echogenic hilum, calcification, cystic component, or disorganized vascularity on US are suggestive of recurrence.

ATA low-risk and intermediate-risk patients, after remnant ablation or adjuvant therapy and negative neck US, should get the serum Tg estimated at 6–18 months on thyroxine therapy [14]. In postoperative MTC patients with a serum calcitonin level of <150 pg/mL, physical examination and US of the neck should be performed, and if negative, it should be followed up with physical examinations, serum levels of calcitonin and CEA, and 6-monthly USs [19]. If the serum calcitonin level is >150 pg/mL, assessment with neck US, chest CT, CEMRI or three-phase CECT of the liver, bone scintigraphy, and MRI of the pelvis and axial skeleton are warranted [19].

Imaging response assessment of extracerebral metastatic disease for those on systemic therapy should be performed using the latest version of Response Evaluation Criteria in Solid Tumors (RECIST 1.1) after chemotherapy and immune RECIST (iRECIST) after immunotherapy [100,101,102,103,104]. For the evaluation of the response of brain metastasis after chemotherapy, response assessment in neuro-oncology brain metastases (RANO BM) is used [101,105].

## 9. Management of Recurrent Disease

CECT/CEMRI of the neck and upper chest should be performed when there is diffuse bulky recurrent nodal disease, elevated Tg with negative neck US, and invasive recurrent disease with invasion of aerodigestive tract [14]. CECT chest should be performed in high-risk DTC patients with increased serum Tg (>10 ng/mL) or increasing Tg antibodies irrespective of the RAI imaging result [14]. Negative neck and chest examination in high-risk DTC patients with elevated Tg (>10 ng/mL) should be followed by imaging of other organs, such as MRI brain, CECT/CEMRI abdomen, and MR skeletal survey, if the patients are symptomatic for these sites [14]. Therapeutic central and/or lateral compartmental neck dissection in a previously operated compartment should be performed for biopsy-proven persistent or recurrent disease for central neck nodes ≥8 mm and lateral neck nodes ≥10 mm in the smallest dimension [14].

### Recurrence in MTC

There is no single PET-CT imaging tracer that can reveal all MTC recurrences or metastases in patients who present with disease recurrence and raised serum calcitonin levels. Short calcitonin doubling time (≤24.1 months, used by different investigators) correlates with higher ^18^F-FDG avidity in lesions and poorer prognosis [106]. The somatostatin receptor (SSTR)-based PET-CT, such as ^68^Ga-DOTA-TATE/TOC, has evolved as a diagnostic PET radiotracer that shows acceptable sensitivity and, importantly, theranostic applications for determining the patient’s eligibility for peptide receptor radionuclide therapy (PRRT) [107].

## 10. Management of Papillary Microcarcinoma

The 2015 ATA management guidelines for DTC suggest active surveillance (using US) for low-risk (absence of regional nodal or distant metastasis and absence of high-grade histopathological features and extrathyroidal extension infiltrating adjacent structures) PMCs due to their indolent nature and unfavorable cost/benefit ratio [8,14]. Rescue surgery is indicated if there is progression of PMC during observation, such as an increase in size by ≥3 mm or new nodal metastasis [8,108]. There is no role for adjuvant RAI therapy in a case in which a low-risk unifocal or multifocal PMC undergoes surgery, as there is no improvement in disease-specific or disease-free survival [14]. PMC with high-risk features should undergo immediate surgery [14,108].

## 11. Percutaneous Ablation

Percutaneous thermal (radiofrequency, laser, microwave, and high-frequency ultrasound) or chemical (ethanol) ablation may be performed for papillary microcarcinoma in patients unwilling to have active surveillance, refusing surgery, or at high-risk for surgery [109,110]. In addition, recurrent thyroid carcinoma patients with a high surgical risk who are unwilling to have repeat surgery may also be managed using percutaneous ablation techniques [111].

## 12. Imaging Recommendations for Pediatric Thyroid Carcinoma

PTC is the most common thyroid carcinoma in the pediatric population and may present as a diffuse infiltrating disease with a higher propensity for cervical nodal metastasis [112]. An FNA decision for a thyroid nodule in children should be based on risk factors and US characteristics and not merely on nodule size. Surgery is preferred over repeat FNA for nodules with indeterminate cytology [112]. For postoperative surveillance, neck US should be performed after 6 months and then at 6- to 12-month intervals for ATA pediatric intermediate- and high-risk patients and annually for ATA pediatric low-risk patients [112]. A posttreatment WBS with/without SPECT/CT is recommended for all children 4–7 days after ^131^I therapy [109].

There is no uniformity in using cross-sectional imaging for the baseline evaluation of thyroid carcinoma. There is a need for an up-to-date systemic review and a meta-analysis evaluating the role of cross-sectional imaging for thyroid carcinoma management. There is also a need to develop surveillance guidelines for low-risk PMC by the ATA.

## 13. Conclusions

US plays a pertinent role in the detection and localization of thyroid cancer and neck nodal metastasis, guiding FNA, and also for the evaluation of the post-operative thyroid bed, to look for residual or recurrent disease. US is the imaging modality of choice for surveillance. Cross-sectional imaging is indicated when there is suspicion of ETE. FDG-PET CECT has an important role in the detection of distant metastasis and the identification of dedifferentiated DTC. RAI has both diagnostic and therapeutic value.

## Figures and Tables

**Table 1:** Ultrasound-based thyroid nodule risk stratification systems (adapted from Mahajan et al. [40]).

Categories						
TIRADS (Horvath et al. [33])	Normal (0%)	Benign (5%)	Probably benign (<5%)	Suspicious (5–80%) 4a (5–10%) 4b (10–80%)	Probably malignant (>80%)	Biopsy proven malignancy
TIRADS (Kwak et al. [34])	Negative	Benign (0%)	Probably benign (1.7%)	Suspicious 4a (3–30%) 4b (9.2%) 4c (44.4–72.4%)	Highly suggestive of malignancy (87.5%)	
F-TIRADS	Normal	Benign (0%)	Very probably benign (0.25%)	4a: Suspicious, low risk of malignancy (6%) 4b: Suspicious, high risk of malignancy (69%)	Effectively certainly malignant nodules (100%)	
BTA	U1: Normal	U2: Benign	U3: Indeterminate	U4: Suspicious	U5: Malignant	
ATA	Benign (<1%)	Very low suspicion (<3%)	Low suspicion (5–10%)	Indeterminate suspicion (10–20%)	High suspicion (>70–90%)	
K-TIRADS		Category 1: Benign (0%)	Category 2: Probably benign (<5%)	Category 3: Indeterminate (5–50%)	Category 4: Suspicious for malignancy (>50%)	
ACR-TIRADS	TR 1: Benign	TR 2: Not suspicious	TR3: Mildly suspicious	TR4: Moderately suspicious	TR5: Highly suspicious	
TMC-RSS		Group 1: Low risk (2.4%)		Group 2: Intermediate risk (18%)	Group 3: High risk (80%)	
AACE/ACE-AME		Class 1: Low-risk thyroid lesion Malignancy risk: 1%	Class 2: Intermediate-risk thyroid lesion Malignancy risk: 5–15%		Class 3: High-risk thyroid lesion Malignancy risk: 50–90%	
EU-TIRADS	EU-TIRADS 1 (Normal)	EU-TIRADS 2 (Benign category) Malignancy risk: 0% EU-TIRADS 3 (Low-risk category) Malignancy risk: 2–4%	EU-TIRADS 4 (Intermediate-risk category) Malignancy risk: 6–17%	EU-TIRADS 5 (High-risk category) Malignancy risk: 26–87%		

American Thyroid Association (ATA), American College of Radiology Thyroid Imaging Reporting and Data Systems (ACR TI-RADS), TI-RADS released by the Korean Society of Thyroid Radiology (K-TIRADS), European Thyroid Association (EU-TIRADS), British Thyroid Association (BTA), TIRADS developed by Kwak et al. [34]. (Kwak-TIRADS), AACE/ACE/AME: American Association of Clinical Endocrinologists/American College of Endocrinology/Associazione Medici Endocrinologi, French (F)-TIRADS, and Thyroid Multimodal-imaging Comprehensive Risk Stratification Scoring (TMC-RSS).

**Table 2:** Studies comparing diagnostic performance of various ultrasound-based thyroid nodule risk stratification systems.

Studies	BTA	EU-TIRADS	K-TIRADS	ACR-TIRADS	Kwak-TIRADS	ATA	AACE/ACE/AME
Grani et al. [39] Best performance by ACR-TIRADS with FNR of 2.2%		Unnecessary FNA avoided in 30.7% S = 86.1% Sp = 32% PPV = 8.9% NPV = 96.7%	Unnecessary FNA avoided in 17.1% S = 91.7% Sp = 17.8% PPV = 7.9% NPV = 96.5%	Unnecessary FNA avoided in 53.4% S = 83.3% Sp = 56.2% PPV = 12.8% NPV = 97.8%		Unnecessary FNA avoided in 34.9% S = 86.1% Sp = 36.5% PPV = 9.5% NPV = 97.1%	Unnecessary FNA avoided in 43.8% S = 75% Sp = 45.3% PPV = 9.6% NPV = 95.9%
Peng et al. [55] Good performance by ACR-TIRADS, ATA, AACE/ACE/AME				S = 94.9% Sp = 58.1% PPV = 76.9% NPV = 88.5% A = 80.0%		S = 92.5% Sp = 68.4% PPV = 79.7% NPV = 87.% A = 82.2%	S = 88.3% Sp = 75.3% PPV = 84.0% NPV = 81.4% A = 83.0%
Chng et al. [56] BTA, Kwak-TIRADS, and ATA have high S and NPV.	S = 90% Sp = 50.9% PPV = 45.5% NPV = 91.8%				S = 94% Sp = 28.2% PPV = 37.3% NPV = 91.2%	S = 98% Sp = 17.3% PPV = 35% NPV = 95%	
Shen et al. [57] Good diagnostic performances by EU-TITADS, ACR-TIRADS, Kwak-TIRADS, and ATA (AUCs > 86%).		S = 93.4% Sp = 81.1% PPV = 81.9% NPV = 92.9%		S = 88.2% Sp = 87.5% PPV = 86.7% NPV = 89%	S = 93.5% Sp = 85.8% PPV = 86% NPV = 93.4%	S = 91.7% Sp = 82% PPV = 82.4% NPV = 92.9%	
Xu et al. [58] -Lowest rate of unnecessary FNA by ACR-TIRADS -Most effective diagnostic performance in specificity by K-TIRADS		S = 83.2% Sp = 79.4% PPV = 73.5% NPV = 87.3%	S = 71.4% Sp = 87.4% PPV = 79.6% NPV = 81.6%	S = 96.6% Sp = 52.9% PPV = 58.6% NPV = 95.8%			
Marukatat et al. [59] Similar results by K-TIRADS and EU-TIRADS for predicting malignancy		S = 86.2% Sp = 75.5% PPV = 56.6% NPV = 93.7%	S = 83.5% Sp = 76.8% PPV = 57.8% NPV = 93.4%				

ATA: American Thyroid Association, ACR-TIRADS: American College of Radiology Thyroid Imaging Reporting and Data Systems, K-TIRADS: TIRADS released by the Korean Society of Thyroid Radiology, EU-TIRADS: European Thyroid Association TIRADS, BTA: British Thyroid Association, Kwak-TIRADS: TIRADS developed by Kwak et al. [34], AACE/ACE/AME: American Association of Clinical Endocrinologists/American College of Endocrinology and Associazione Medici Endocrinologi, FNA: Fine needle aspiration, FNR: False Negative Rate, S: Sensitivity, Sp: Specificity, PPV: Positive Predictive Value, NPV: Negative Predictive Value, A: Accuracy.

**Table 3:** Imaging guidelines for staging medullary and anaplastic thyroid carcinomas based on the American Thyroid Association recommendations.

Type of Thyroid Carcinoma	Imaging Recommendations
Medullary thyroid carcinoma	CECT of the neck and chest, three-phase CECT of the liver or CEMRI of the liver, and axial MRI and bone scintigraphy are recommended for those with widespread disease in the neck along with signs or symptoms of regional or distant metastases and also in those with a serum calcitonin of more than 500 pg/mL.
Anaplastic thyroid carcinoma	FDG-PET/CT with or without CECT is recommended for staging, otherwise CEMRI of neck, chest, abdomen, and pelvis is required. CEMRI brain to be performed at initial staging and when clinically indicated. In the absence of a PET scan, bone scan should be performed to identify bone metastasis in ATC. In addition, laryngoscopy with or without esophagoscopy and bronchoscopy may be considered as part of staging for ATC.

CECT: Contrast-Enhanced Computed Tomography, CEMRI: Contrast-Enhanced Magnetic Resonance Imaging, FDG-PET/CT: ^18^F-fluorodeoxyglucose positron emission tomography/CT.

**Table 4:** Adjacent structure encasement criteria for thyroid carcinoma on CT/MRI.

Author	Trachea	Esophagus	CCA/IJV/Vessel	RLN
Wang et al. [74]		Outer layer encasement		
Seo et al. [76]	One of the following on CT: ≥180° contact Deformity of the tracheal lumen at the level of the mass. Focal irregularity, thickening, or bulge in the mucosa adjacent to the mass.	One of the following on CT: ≥180° contact Loss of normal esophageal wall and lumen.	≥180° contact	Two of the following: Completely effaced fat in the tracheoesophageal groove >25% abutment at the posterior portions of the thyroid (posterior extracapsular invasion) Ipsilateral vocal cord palsy on CT seen as paramedian cord, anteromedial deviation of the arytenoid cartilage, enlarged pyriform sinus, or enlarged laryngeal ventricle
Mancuso et al. [77]			≥90° contact	
Takashima et al. [78]	≥180° contact	≥180° contact	≥180° contact	
Takashima et al. [79]				Effaced fat in the tracheoesophageal groove
Ishikawa et al. [80]	≥180° contact	≥180° contact	≥180° contact	

CCA: Common carotid artery, IJV: Internal jugular vein, RLN: Recurrent laryngeal nerve.

**Table 5:** Imaging findings and their implications in the management of thyroid carcinomas (adapted from Mahajan et al. [71]).

Imaging Findings	Implications in Thyroid Carcinoma Management
ETE infiltrating strap muscles	-Total thyroidectomy with/without RAI -Complete resection without reconstruction.
Tracheal invasion as per SHIN grading (Figure 3)	-Total thyroidectomy with/without RAI -Grade 1: Shave procedure without any residual disease -Remaining grades: window resection/circumferential tracheal resection and re-anastomosis.
Soft tissue in tracheoesophageal groove suggesting RLN involvement with vocal cord paralysis	-Total thyroidectomy with/without RAI -RLN resected only if soft tissue adherent to RLN and its function is compromised preoperatively.
Esophageal encasement > 180 degree or frank infiltration	-Total thyroidectomy with/without RAI -Extensive involvement till mucosa and submucosa: Segmental resection with flap reconstruction -Involvement of only muscularis layer: Margins possible without segmental resection.
Involvement of larynx	-Total thyroidectomy with RAI -Extensive involvement: Partial/total laryngectomy -Superficial involvement: Shave excision.
Prevertebral fascia infiltration	Total thyroidectomy with RAI.
Encasement of carotid artery by >270 degree	Total thyroidectomy with RAI.
Aberrant right subclavian artery suggesting non-recurrent inferior laryngeal nerve	-Total thyroidectomy with/without RAI -Careful dissection to preserve the nerve.
Internal jugular vein tumor thrombosis/involvement	-Total thyroidectomy with RAI -If bilateral IJV involved, then resected with reconstruction, provided adequate proximal and distal stump present. -If unilateral IJV involved, then resected without reconstruction, provided adequate proximal and distal stump present.
Nodal burden	-No nodes: only total thyroidectomy -Nodal involvement: Total thyroidectomy with RAI + neck dissection, including central compartment clearance.
Distant metastasis	-Total thyroidectomy with RAI.

ETE: Extrathyroidal extension, RLN: Recurrent laryngeal nerve, IJV: Internal jugular vein, RAI: radioactive iodine.

**Table 6:** Studies predominantly using cross-sectional imaging for baseline evaluation of thyroid carcinoma and neck nodes.

Authors	Nature of Study	Sample Size	Imaging Modality	Outcome	Comments
Alabousi et al. (2022) [48]	Systematic review	31,942	CT and US	For central compartment nodes: CT S = 39% Sp = 87% US S = 28% Sp = 95% For lateral compartment nodes: CT S = 77% Sp = 88% US S = 73% Sp = 89% *For ETE:* CT and US: S = 86–91% Sp = 30–47%	CT was more sensitive for central compartment neck nodal metastasis, whereas US was more specific. No significant difference in the diagnostic accuracy for lateral compartment neck nodal metastasis between US and CT.
Seo et al. (2010) [76]	Diagnostic accuracy for ETE	84	CT	For tracheal invasion: S = 59.1%, Sp = 91.4%, A = 83.2% For esophageal invasion: S = 28.6%, Sp = 96.2%, A = 90.7% For invasion of CCA: S = 75.0%, Sp = 99.4%, A = 98.8% For invasion of IJV: S = 33.3%, Sp = 98.8%, A = 97.1% For invasion of RLN: S = 78.2%, Sp = 89.8%, A = 85.5%	Despite a low sensitivity, CT can be a valuable modality for ETE detection.
Shalash et al. (2022) [84]	Comparative study for detection of cervical nodal metastasis in DTC	30	DW-MRI and 18F-FDG PET/CT	PET-CT S = 84% Sp = 80% NPV = 50% PPV = 95% A = 83% DW-MRI S = 84% Sp = 60% NPV = 42.8% PPV = 91.3% A = 80% Combined PET-CT and DW-MRI S = 96% NPV = 80%	18F-FDG PET/CT outperforms DW-MRI for the assessment of neck nodal deposits.
Zhang et al. (2020) [85]	Diagnostic study for PTC	82	CT	S = 87.8%, Sp = 94.2%, A = 91.1%	Irregular ring, marginal defects, and enhanced blurring on CT were strongly correlated with PTC.
Hu et al. (2020) [86]	Comparative study for ETE in PTC	225	MRI and US	For minimal ETE: MRI S = 71.3% Sp = 77.1% PPV = 83.8% NPV = 61.7% A = 73.4% US S = 87.5% Sp = 66.6% PPV = 81.4% NPV = 76.2% A = 79.7% For extensive ETE: MRI S = 85.4% Sp = 76.2% PPV = 68.3% NPV = 89.7% A = 79.7% US S = 87.5% Sp = 66.6% PPV = 81.4% NPV = 76.2% A = 79.7% Overall ETE: MRI S = 76.6% Sp = 93.8% PPV = 89.1% NPV = 85.7% A = 86.9% US S = 79.7% Sp = 83.3% PPV = 76.1% NPV = 86% A = 81.9%	For minimal ETE prediction: preoperative US should be used as the first-line imaging. For extensive ETE evaluation: MRI should be added. For overall ETE: MRI had higher specificity and PPV than US.
Cho et al. (2020) [87]	Systematic review and meta-analysis	504	MRI	S = 80% Sp = 85%	Moderate diagnostic performance of MRI for neck nodal metastasis in thyroid cancer. May be an optional or complementary imaging method to US or CT.

PTC: Papillary thyroid carcinoma, DTC: Differentiated thyroid cancer, MRI: Magnetic resonance imaging, DW-MRI: Diffusion-weighted MRI, US: Ultrasound, CT: Computed tomography, FDG PET: Fluorodeoxyglucose positron emission tomography, S: Sensitivity, Sp: Specificity, A: Accuracy PPV: Positive Predictive Value, NPV: Negative Predictive Value, A: Accuracy, IJV: Internal jugular vein, CCA: Common carotid artery, RLN: Recurrent laryngeal nerve.

## Supplementary Materials

The following supporting information can be downloaded at: https://www.mdpi.com/article/10.3390/jcm13102904/s1, Table S1: Eighth edition American Joint Committee on Cancer (AJCC) Tumor, Regional Lymph Node and Distant Metastasis (TNM) descriptors for differentiated, medullary, and anaplastic thyroid cancer, Table S2: Stage groups for differentiated thyroid cancer (DTC), anaplastic thyroid cancer (ATC), and medullary thyroid cancer (MTC), Figure S1: Synoptic reporting template for thyroid carcinoma on contrast-enhanced computed tomography (CECT).

## Abbreviations

HNC	Head and neck cancer
PTC	Papillary thyroid carcinoma
FTC	Follicular thyroid carcinoma
MTC	Medullary thyroid carcinoma
PDTC	Poorly differentiated thyroid carcinoma
ATC	Anaplastic thyroid carcinoma
WHO	World Health Organization
PMC	Papillary microcarcinoma
CT	Computed tomography
MRI	Magnetic resonance imaging
FDG PET	Fluorodeoxyglucose positron emission tomography
RT	Radiation therapy
MEN	Multiple endocrine neoplasia
MAPK	Mitogen-activated protein kinase
PI3K	Phosphatidylinositol-3 kinase
RAS	Rat sarcoma virus
PTEN	Phosphatase and tensin homolog
ETE	Extrathyroidal extension
ATA	American Thyroid Association
AACE	American Association of Clinical Endocrinologists
NCCN	National Comprehensive Cancer Network
ESMO	European Society of Medical Oncology
FNA	Fine needle aspiration
ACR TI-RADS	American College of Radiology Thyroid Imaging Reporting and Data Systems
K-TIRADS	Korean Society of Thyroid Radiology Thyroid Imaging, Reporting and Data Systems
EU-TIRADS	European Thyroid Association
BTA	British Thyroid Association
SRU	Society of Radiologists in Ultrasound
ACE	American College of Endocrinology
AME	Associazione Medici Endocrinologi
F-TIRADS	French-TIRADS
TMC-RSS	Thyroid Multimodal-imaging Comprehensive Risk Stratification Scoring
TSH	Thyrotropin
US	Ultrasound
CEA	Carcinoembryonic antigen
IHC	Immunohistochemistry
ENE	Extranodal extension
Tg	Thyroglobulin
TNM	Tumor, Node, Metastasis
AJCC	American Joint Committee on Cancer
UICC	Union for International Cancer Control
CECT	Contrast-enhanced computed tomography
WBS	Whole body scintigraphy
SNMMI	Society of Nuclear Medicine and Molecular Imaging
EANM	European Association of Nuclear Medicine
SUV	Standardized uptake value
MTV	Metabolic tumor volume
TLG	Total lesion glycolysis
RLN	Recurrent laryngeal nerve
EBSLN	External branch of the superior laryngeal nerve
EBRT	External beam radiotherapy
IMRT	Intensity modulated radiotherapy
SPECT	Single photon emission computed tomography
RECIST	Response Evaluation Criteria in Solid Tumors
iRECIST	Immune RECIST
RANO BM	Response assessment in neuro-oncology brain metastases
SSTR	Somatostatin receptor
PRRT	Peptide receptor radionuclide therapy
