# Supplementary material for: Comprehensive Review of the Imaging Recommendations for Diagnosis, Staging, and Management of Thyroid Carcinoma
Source: J Clin Med. 2024 May 14;13(10):2904. doi: 10.3390/jcm13102904 (PMC11122658; doi:10.3390/jcm13102904)
Supplement: Supplementary file 1 [file jcm-13-02904-s001.zip › jcm-2995393-supplementary.pdf]

Table S1: Eight edition American Joint Committee on Cancer (AJCC) Tumour, Regional Lymph Node and Distant Metastasis (TNM) descriptors for differentiated, medullary, and anaplastic thyroid cancer.

**T descriptor**	
Tx	Primary tumor cannot be assessed
T0	No evidence of primary tumor is found
T1	Tumor size ≤ 2 cm in greatest dimension and is limited to the thyroid
T1 a	Tumor ≤ 1 cm, limited to the thyroid
T1 b	Tumor > 1 cm but ≤ 2 cm in greatest dimension, limited to the thyroid
T2	Tumor size > 2 cm but ≤ 4 cm, limited to the thyroid
T3	Tumor size > 4 cm, limited to the thyroid or any tumor with gross extrathyroidal extension invading only strap muscles
T3 a	Tumor size > 4 cm, limited to the thyroid
T3 b	Any size tumor with gross extrathyroidal extension invading only strap muscles (e.g., extension to sternothyroid, sternohyoid, thyrohyoid, or omohyoid muscles)
T4 a	Any size tumor with gross extrathyroidal extension invading subcutaneous soft tissues, larynx, trachea, esophagus, or recurrent laryngeal nerve
T4 b	Any size tumor with gross extrathyroidal extension invading prevertebral fascia or encasing the carotid artery or mediastinal vessels
**N descriptor**	
NX	Regional nodes cannot be assessed
N0	No regional lymph node metastasis
N0a	One or more cytologically or histologically confirmed benign lymph nodes
N0b	No radiologic or clinical evidence of locoregional lymph node metastasis

N1	Regional lymph node metastasis
N1a	Metastases to level VI or VII (pretracheal, paratracheal, or prelaryngeal/Delphian or upper mediastinal) lymph nodes; can be unilateral or bilateral disease
N1b	Metastases to unilateral, bilateral, or contralateral neck lymph nodes (levels I, II, III, IV, or V) or retropharyngeal lymph nodes
**M descriptor**	
M0	No distant metastasis is found
M1	Distant metastasis is present

Table S2: Stage groups for Differentiated thyroid cancer (DTC), Anaplastic thyroid cancer (ATC) and Medullary thyroid cancer (MTC)

**Stage**	**T**	**N**	**M**
Differentiated thyroid cancer (DTC)			
If age at diagnosis <55			
I	Any T	Any N	M0
II	Any T	Any N	M1
If age at diagnosis ≥55 y:			
I	T1, T2	N0, Nx	M0
II	T1, T2	N1	M0
	T3	Any N	M0
III	T4a	Any N	M0
IV A	T4b	Any N	M0
IV B	Any T	Any N	M1
Anaplastic thyroid cancer (ATC)			
IV A	T1, T2, T3a	N0, Nx	M0
IV B	T1, T2, T3a	N1	M0
	T3b, T4	Any N	M0
IV C	Any T	Any N	M1
Medullary thyroid cancer (MTC)			
I	T1	N0	M0
II	T2, T3	N0	M0
III	T1, T2, T3	N1 a	M0

**Stage**	**T**	**N**	**M**
IV	T1, T2, T3	N1 b	M0
	T4	Any N	M0
	Any T	Any N	M1

Figure S1: Synoptic reporting template for thyroid carcinoma on contrast enhanced computed tomography (CECT)

Primary Thyroid nodule

Location: Right lobe/Left lobe/Isthmus
Size:
Enhancement: Homogeneous/Heterogeneous
Calcifications: Absent/Present
If present: microcalcification/ macrocalcification/ eggshell
Cystic / Necrotic change: Absent/Present
Extra-thyroid extension: Absent/Present
If present CT Grade of extrathyroid extension (ETE)*:
Mediastinal extension: Absent/Present
Right aberrant subclavian artery: Absent/Present

T stage
Strap muscle involvement: yes/no
T-E groove: Not involved/Involved (Status of vocal cords, indirect sign of RLN involvement)
Relationship with trachea (SHIN grade #):
Fat planes with oesophagus: Lost/ maintained. If lost; angle of contact:
Planes with prevertebral fascia: Lost/ maintained
Cricophraynx: Not involved/Involved
Cricoid cartilage: Not involved/Involved
Angle of contact with CCA (<180 / 180-270/>270):
Angle of contact with innominate vessels (<180 / 180-270/>270):

N Stage
Laterality- Ipsilateral / contralateral / Bilateral
Compartment: central/ lateral
Node stations:

Right cervical nodes
Levels: Level ILevel II Level III Level IV Level V Level VI
Size
Heterogeneity
Calcification
Cystic or necrotic change
Suspicious/ indeterminate/ benign @
Left cervical nodes
Levels: Level ILevel II Level III Level IV Level V Level VI
Size
Heterogeneity
Calcification
Cystic or necrotic change
Suspicious/ indeterminate/ benign @
Vascular involvement:
CCA abutment: Absent/Present
ICA abutment: Absent/Present
ECA abutment: Absent/Present
If present angle of contact for CCA and ICA: <90, 90 – 179, 180 – 269; >270
Strap muscles involvement: Absent/Present
Prevertebral fascia invasion: Absent/Present

M Stage
Lung nodules: Absent / Present
If present:
solitary/ multiple
location:
Size:
suspicious/ TSTC@/ Benign

Any other metastatic lesion (hepatic, skeletal): Absent / Present
If yes, specify location and size:

Impression:
T stage
N stage
M stage

Specific comments, if any:
@ Follow-up/ image guided FNAC correlation.

*CT ETE grading:
•0: a tumor which was completely enveloped by thyroid parenchyma;
•I: a tumor in which the percentage of the tumor perimeter in contact with the thyroid capsule was 1–25%;
•II: a tumor in which the contact with the capsule was 25–50%;
•III: a tumor in which the contact with the capsule was >50%

CT Shin grading:
•0: > 5mm distance between tumor and trachea.
•I: disease abuts external perichondrium.
•II: disease invades into the cartilage +/- destruction.
•III: disease extends into the tracheal mucosa with no elevation/penetration of mucosa.
•IV: disease is full-thickness invasion with expansion of the tracheal mucosa with a bulge.
